# Supplementary material for: Knowledge, attitude and purchasing behavior of Saudi mothers towards food additives and dietary pattern of preschool children
Source: PeerJ. 2024 Oct 10;12:e18223. doi: 10.7717/peerj.18223 (PMC11471143; doi:10.7717/peerj.18223)
Supplement: Supplemental Information 2 [file peerj-12-18223-s002.pdf]

استبانة حول المعرفة والمواقف والسلوك الشرائي للأمهات السعوديات اتجاه المضافات الغذائية والنمط الغذائي للأطفال في سن ما قبل المدرسة

الخصائص الديموغرافية

1- عمر الام:

- من ١٨-٢٢ سنة
- من ٢٣-٢٧ سنة
- من ٢٨-٣١ سنة
- من ٣٢-٣٦ سنة
- من ٣٧- ٤١ سنة
- من ٤٢ – ٤٦ سنة
- أكبر من ٤٦ سنة

2- المستوى التعليمي للأم:

- أقل من الثانوي
- ثانوي
- دبلوم
- بكالوريوس
- دراسات عليا (ماجستير- دكتوراه)

3- الحالة الاجتماعية للأم:

- متزوجة
- مطلقة
- أرملة

4- الحالة الوظيفية للأم:

- ربة منزل
- طالبة

- موظفة
- اعمل لحسابي الخاص (أعمال حرة)
- متقاعدة

5- الحالة الاقتصادية للعائلة (الدخل الشهري):

- أقل من ٤٠٠٠ ريال سعودي شهرياً
- ٤٠٠٠ – ٨٠٠٠ ريال سعودي شهرياً
- ٩٠٠٠- ١٥,٠٠٠ ريال سعودي شهرياً
- ١٦،٠٠٠- ٢٥,٠٠٠ ريال سعودي شهرياً
- أكثر من ٢٥,٠٠٠ ريال سعودي شهرياً

6- المدينة التي تعيش فيها الأم (في المنطقة الغربية):

- جدة
- مكة المكرمة
- المدينة المنورة
- الطائف
- ينبع
- أخرى (............)

7- عدد الأطفال في سن ما قبل المدرسة (من عمر ٣ – ٥ سنوات):

- ١
- ٢
- ٣
- أكثر من ٣

8- كم عمر طفلك؟ (إذا كان لديك أكثر من طفل واحد من 3 إلى 5 سنوات، فاختر الطفل الأكبر للإجابة على هذا الاستبانة):

- ٣ سنوات

- ٤ سنوات
- ٥ سنوات

9- وزن الطفل بالكيلو جرام (كجم):................

10- طول الطفل بالسنتيمتير (سم):.............

11- هل يعاني طفلك من الحساسية لبعض الأطعمة؟

- نعم
- لا
- لا اعلم

12- هل يعاني طفلك من تسوس الاسنان؟

- نعم
- لا
- لا اعلم

المحور الأول: معرفة الأمهات بالمضافات الغذائية

1- هل سمعت عن المضافات الغذائية (Food Additives) من قبل؟

- نعم
- لا

2- إذا كانت إجابتك بنعم، ما هو مصدر معلوماتك عن المضافات الغذائية؟

- وزارة الصحة / هيئة الغذاء والدواء
- المدارس / الجامعات
- مواقع التواصل الاجتماعي / الأنترنت

- التلفاز / الراديو
- الصحف والمجلات
- المؤتمرات والندوات
- العائلة والأصدقاء
- أخرى

3- المضافات الغذائية (Food Additives) هي مواد مصنعة تضاف إلى الأغذية لتحسين نكهتها ولونها وملمسها ولزيادة مدة حفظها:

- نعم
- لا
- لا أعلم

4- تُصنف المضافات الغذائية في الأطعمة كمواد حافظة، ملونات، مضادات أكسدة ومستحلبات:

- نعم
- لا
- لا أعلم

5- تعتمد مصانع الأغذية على معايير عالمية لتحديد النسب القصوى المسموح بها من المضافات الغذائية لاستخدامها في منتجاتها:

- نعم
- لا
- لا أعلم

6- تضع مصانع الأغذية في المملكة العربية السعودية قائمة واضحة بالمواد المضافة في منتجاتها الغذائية:

- نعم
- لا
- لا اعلم

7- تُكتب في البطاقة الغذائية الإرشادية المضافات الغذائية الموجودة في المنتج وبعض المضافات الغذائية يرمز لها بالحرف(E):

- نعم
- لا
- لا أعلم

8- ترمز الأرقام بعد حرف E مثل (E129) إلى نوع المضافات الغذائية الموجودة في المنتج:

- نعم
- لا
- لا أعلم

9- تنقسم المضافات الغذائية في الغذاء المصنع الى مضافات طبيعية وصناعية:

- نعم
- لا
- لا أعلم

10- قد تسبب زيادة تناول المضافات الغذائية الصناعية فرط نشاط لدى الأطفال وأمراض أخرى خطيرة:

- نعم
- لا
- لا اعلم

المحور الثاني: مواقف (معتقدات) الأم من المضافات الغذائية

1- هل تسمحي لطفلك بتناول أطعمه تحتوي على مضافات غذائية؟

- نعم
- لا
- لا أهتم

2- هل لديك معلومات غذائية كافية عند اختيار المنتج الغذائي المناسب وما يضاف اليه من مضافات غذائية؟

- نعم
- لا
- ربما

3- الغذاء الذي يحتوي على المضافات الغذائية الصناعية أكثر جذباً للمستهلك:

- نعم
- لا
- لا أعلم

4- إن المضافات الغذائية في المنتجات الغذائية والمعتمدة من المملكة العربية السعودية آمنه صحياً؟

- نعم
- لا
- لا أعلم

5- لدي قلق من احتمالية حدوث أمراض بعد تناول أطعمة تحتوي على مضافات غذائية:

- نعم
- لا
- أحيانا

6- لدي خوف من إصابة طفلي بالحساسية بعد تناول أطعمة تحتوي على مضافات غذائية:

- نعم
- لا
- أحيانا

7- لدي استعداد لدفع مال أكثر لشراء منتج غذائي خالي من مضافات غذائية:

- نعم
- لا
- ربما

8- هل تعتقد أنه من الضروري إعداد برامج توعية للمجتمع حول المضافات الغذائية للأطعمة:

- نعم

- لا
- لا اهتم

المحور الثالث: السلوك الشرائي للأم

1- اطلع جيدا على الملصق الغذائي للمنتج لمعرفة المكونات الغذائية وما به من مضافات غذائية قبل شراء أطعمة أطفالي:

- دائماً
- أحياناً
- نادراً
- أبداً

2- أتجنب شراء المنتجات الغذائية التي تحتوي على نسبة عالية من السكريات لأطفالي مثل العصائر المعلبة:

- دائماً
- أحياناً
- نادراً
- أبداً

3- أتجنب شراء المنتجات الغذائية التي تحتوي على نسبة عالية من الألوان لأطفالي مثل حلوى المصاص والسكاكر:

- دائماً
- أحياناً
- نادراً

- أبداً

4- أتجب شراء المنتجات الغذائية التي تحتوي على نكهات عالية لأطفالي مثل رقائق البطاطس(الشبس):

- دائماً
- أحياناً
- نادراً
- أبداً

5- أتجنب شراء المنتجات الغذائية التي تحتوي على محسن التوابل والنكهة (الملح الصيني "اجينوموتو") لأطفالي مثل الشوربة سريعة التحضير:

- دائماً
- أحياناً
- نادراً
- أبداً

6- أحرص على اختيار المنتجات الطازجة الخالية من المضافات الغذائية لأطفالي:

- دائماً
- أحياناً
- نادراً
- أبداً

7- اسمح لأطفالي بشراء ما يرغبون به من مأكولات ومشروبات:

- دائماً
- أحياناً
- نادراً
- أبداً

8- لا أستشير أطفالي عند شرائي للأطعمة:

- دائماً
- أحياناً
- نادراً
- أبداً

النمط الغذائي لأطفال ما قبل المدرسة:

الأطعمة	أكثر من ١ في اليوم	١ في اليوم	٤-٥ مرات في الاسبوع	٢-٣ مرات في الأسبوع	مرة واحدة أسبوعياً	مره أو أقل في الشهر	أبداً
**البسكويت والمقرمشات** (مقرمشات الذرة والقمح والبسكويت بأنواعه)							
**رقائق البطاطس** (الشبس بأنواعه مثل الشيتوس، ليز، دوريتوس وغيره)							
**الكيك** (بان كيك، تشيزكيك، كيك الشكولاتة وغيره)							
**حبوب الإفطار** (الكورن فليكس بأنواعه)							
**منتجات الألبان المنهكة** (زبادي بالتوت، حليب الشكولاتة وغيره)							
**العلكة** (العلكة الفقاعية والكروية وغيرها)							
**العصائر** (العصائر المعلبة مثل السن توب، نكتار وغيره)							
**المشروبات الغازية** (بيبسي، سفن اب وغيره)							

**الحلويات** (حلوى المصاص وغيرها)							
**الشكولاتة** (بأنواعها)							
**الآيس كريم** (كواليتي، السعودية توت، فواكه، شكولاتة وغيره)							
**الجلي** (الجلي الحلو، الحامض)							
**اللحم المصنع** (نقانق، مارتديلا، ناجت الدجاج)							
**الصلصات** (المايونيز، الكاتشب، صلصة الرانش وغيره)							
**الشعرية والشوربة سريعة التحضير** (اندومي ، شوربة ماجي وغيره)							
