# Supplementary material for: Knowledge, attitude and purchasing behavior of Saudi mothers towards food additives and dietary pattern of preschool children
Source: PeerJ. 2024 Oct 10;12:e18223. doi: 10.7717/peerj.18223 (PMC11471143; doi:10.7717/peerj.18223)
Supplement: Supplemental Information 3 [file peerj-12-18223-s003.pdf]

Survey about Knowledge, Attitude and Purchasing Behavior of Saudi Mothers Towards Food Additives and Dietary Pattern of Preschool Children:

❖ Demographic characteristics:

1- Age of mother:
- 18 – 22 years old
- 23 – 27 years old
- 28 – 31 years old
- 32 – 36 years old
- 37 – 41 years old
- 42 – 46 years old
- Above 46 years old

2- Mother's education level:
- Less than high school
- High school
- Diploma
- Bachelor
- Postgraduate studies

3- Marital status of mother:
- Married
- Divorced
- Widow

4- Mother Occupation status:
- Businesswoman
- Employee
- Housewife
- Students
- Retired

5- Economic situations:
- Under 4,000 SR per month
- 4,000 – less than 8,000 SR per month
- 8,000 –less than 15,000 SR per month

- 15,000 – less than 25,000 SR per month
- Above 25,000 SR per month

6- The city you live (in west region):
- Makkah city
- Medina city
- Jeddah city
- Yanbu city
- Taif city
- Others

7- Number of preschool children (from 3 to 5 years):
- 1
- 2
- 3
- More than 3

8- How old is your child? If you have more than one child from 3 to 5 years old, choose the oldest child to answer this survey:
- 3 years
- 4 years
- 5 years

9- Child's weight in kilograms (kg):
……………

10- Child's height in centimeters (cm):
………….

11- Does your child suffer from food allergies?
- Yes
- No
- I don't know

12- Does your child suffer from tooth decay?
- Yes
- No
- I don't know

❖ Mothers' knowledge toward food additives:

1- Have you ever heard about food additives?
    - Yes
    - No
    - I don't know

2- If you answer yes, what are your sources of information about food additives?

- Ministry of Health \ Food and Drug Administration
- Universities \ schools
- Social media \ Internet
- TV/radio
- Newspapers and magazines
- Conferences and symposia
- Family and friends
- Others

3- Food additives are materials added to processed food to improve the flavor, color, texture and to extend preservation period:
- Yes
- No
- I don't know

4- The types of additives in foods are preservatives, coloring agents, antioxidants, and emulsifiers:
- Yes
- No
- I don't know

5- Food factories adhere to certain regulations that are globally specific to the proportions of food additives authorized to be used in their products:
- Yes
- No
- I don't know

6- Food factories in Saudi Arabia put a clear list of additives in their food products:
- Yes

- No
- I don’t know

7- Do you k0w that the kind of food additives be written in the guidance card on the package and symbolized by the letter E?
- Yes
- No
- I don’t know

8- The numbers after the letter E, such as (E129), symbolize the type of food additive present in the product:
- Yes
- No
- I don’t know

9- Food Additives in processed food products are divided into natural and artificial additives
- Yes
- No
- I don’t know

10- High consumption of artificial food additives may cause hyperactivity for children and other dangerous disease
- Yes
- No
- I don’t know

❖ Mothers’ attuited toward food additives:

1- Do you allow your child to eat foods that contain food additives?
  - Yes
  - No
  - I don't care

2- Do you have enough nutritional information to select the suitable food product including food additives?
  - Yes
  - No
  - Maybe

3- Food containing artificial additives is more attractive for consumers:
- Yes
- No
- I don't know

4- The approved food additives in food products in Saudi Arabia are safe of health?
- Yes
- No
- I don’t know

5- I am worried for my child to have diseases after consumption food with additives:
- Yes
- No
- Sometime

6- I am afraid for my child to get allergy after consumption food with additives:
- Yes
- No
- Sometime

7- Are you willing to pay more money for food without additives?
- Yes
- No
- Maybe

8- Do you think that it is essential to prepare awareness programs for our community about food additives?
- Yes
- No
- I don't care

❖ Mothers purchasing behavior

1- When buying food products for my children, I pay attention to information on food label related to additives as ingredients:
- Always

- Sometime
- Rarely
- Never

2- I avoid purchasing food products that contain high sweeteners for my children such as canned juices :

- Always
- Sometime
- Rarely
- Never

3- I avoid purchasing food products that contain high colorings for my children such as lollipops sweet and candies:

- Always
- Sometime
- Rarely
- Never

4- I avoid purchasing food products that contain high flavors for my children such as potato chips:

- Always
- Sometime
- Rarely
- Never

5- I avoid purchasing food products that contain monosodium glutamate (MSG) such as instant soup:

- Always
- Sometime
- Rarely
- Never

6- l am keening to select natural products for my children:

- Always
- Sometime
- Rarely
- Never

7- My children can choose any food and beverages they want it :

- Always
- Sometime
- Rarely
- Never

8- I do not consult my children when I buy food:

- Always
- Sometime
- Rarely
- Never

❖ Dietary pattern of preschool children:

Food item	more than once/day	once /day	4-5 /weekly	2-3 weekly	once weekly	once or < monthly	No
**biscuits & crackers** (corn and wheat crackers and all kinds of biscuits)							
**Potato chips** (all kinds of chips such as Cheetos, Lays, Doritos, etc.)							
**Cakes** (Pancake, cheesecake, chocolate cake)							
**Breakfast cereals** (all kinds of corn flakes)							
**Fortified dairy products** (yogurt with berries, chocolate milk, etc.)							
**Chewing gums** (Bubble gum, ball gum, etc)							
**Juices** (canned juices such as Suntop, Nectar, etc.)							
**Soft beverages** (Pepsi, 7UP, etc.)							
**Candies** (lollipops, etc.)							
**Chocolate** (of all kinds)							
**Ice-cream** (Quality, Saudia, berries, fruits, chocolate, etc.)							
**Jelly** (sweet or sour jelly)							

**Processed meat** (sausage, mortadella, chicken nuggets)							
**Sauces** (mayonnaise, ketchup, ranch dressing, etc.)							
**Instant noodles and soups** (Indomie, Maggi, etc.)							
